# Using primary care data to evaluate the 10-year cost-effectiveness of cardiovascular disease risk algorithms in patients with serious mental illness: a patient level simulation

**DOI:** 10.1186/1745-6215-16-S2-P28

**Published:** 2015-11-16

**Authors:** Ella Zomer, Rachael Hunter, Irwin Nazareth, David Osborn

**Affiliations:** 1 University College London, London, UK

## Background

Patients with serious mental illness (SMI) are at increased risk of cardiovascular disease (CVD), but there is limited evidence on the cost-effectiveness of SMI specific CVD risk management strategies.

## Aim

To develop a 10 year patient level simulation of the cost-effectiveness of an SMI-specific CVD risk algorithm compared to standard CVD risk algorithm for primary CVD prevention.

## Methods

Patient data was extracted from The Health Improvement Network (THIN), a primary care database, to populate the patient level simulation. Patients had SMI, were aged 30 to 74 years and free of CVD. A CVD risk algorithm was applied and patients scoring above 10% prescribed statins. We tested four CVD risk algorithms against no algorithm; lipid and body mass index (BMI) versions of SMI specific and general population algorithms. Time dependent transition probabilities for fatal and non-fatal CVD events were calculated using survival analysis. Utility scores to calculate quality adjusted life years (QALYs) were obtained from the literature.

## Results

All four risk algorithms plus statins for those at high risk resulted in more QALYs for less cost than no risk algorithm. The general population lipid and the SMI BMI algorithm had the highest probability of being cost-effective, resulting in an additional 12 QALYs and a cost saving of £37,310 and £36,431 respectively, per 1000 patients over 10 years compared to no algorithm.

## Conclusion

Patient level simulations using primary care data provide a mechanism for assessing the cost-effectiveness of CVD risk management strategies for high risk populations where there is limited evidence.

